# The Inaugural Australian Centre for Hepatitis Virology Public Panel Discussion on Viral Hepatitis Research—Lessons in Scientific Community Outreach

**DOI:** 10.3390/v13091838

**Published:** 2021-09-15

**Authors:** Thomas Tu, Chaturaka Rodrigo, Simone I Strasser, D Scott Bowden, Jennifer H MacLachlan, Heidi E Drummer

**Affiliations:** 1 Australian Centre for Hepatitis Virology, Melbourne, VIC 3000, Australia; c.rodrigo@unsw.edu.au; 2 Centre for Infectious Diseases and Microbiology, Marie Bashir Institute for Infectious Diseases and Biosecurity, University of Sydney at Westmead Hospital, Westmead, NSW 2145, Australia; 3 Storr Liver Centre, Westmead Clinical School and Westmead Institute for Medical Research, Faculty of Medicine and Health, The University of Sydney, Westmead, NSW 2145, Australia; 4 Kirby Institute, UNSW Sydney, Randwick, NSW 2052, Australia; 5 Department of Pathology, School of Medical Sciences, UNSW Sydney, Sydney, NSW 2052, Australia; 6 AW Morrow Gastroenterology and Liver Centre, Royal Prince Alfred Hospital, Camperdown, NSW 2050, Australia; simone.strasser@health.nsw.gov.au; 7 Sydney Medical School, University of Sydney, Camperdown, NSW 2050, Australia; 8 Victorian Infectious Diseases Reference Laboratory, Royal Melbourne Hospital, at the Peter Doherty Institute for Infection and Immunity, Melbourne, VIC 3000, Australia; scott.bowden@vidrl.org.au; 9 WHO Collaborating Centre for Viral Hepatitis, Peter Doherty Institute for Infection and Immunity, Melbourne, VIC 3000, Australia; jennifer.maclachlan@mh.org.au; 10 Department of Medicine, University of Melbourne, Parkville, VIC 3010, Australia; 11 Viral Entry and Vaccines Group, Burnet Institute, Melbourne, VIC 3004, Australia; heidi.drummer@burnet.edu.au; 12 Department of Microbiology and Immunology, Peter Doherty Institute for Infection and Immunity, University of Melbourne, Melbourne, VIC 3000, Australia; 13 Department of Microbiology, Monash University, Clayton, VIC 3800, Australia

**Keywords:** hepatitis B, hepatitis C, community outreach, lived experience

## Abstract

Viral hepatitis remains one of the most significant health issues globally, directly responsible for over 1 million deaths each year and affecting almost 300 million people around the world. Scientific research in recent decades has brought about improvements in the lives of people living with chronic viral hepatitis. On the 29 July 2021, the Australian Centre for Hepatitis Virology (ACHV) for the first time held a public educational forum for the general public. The main aim of this event was to inform the affected community about the importance of scientific research and give an overview of upcoming developments in the field. Here, we provide a detailed report of the panel discussion (including its organisation, execution, and lessons learned to incorporate into future events) and provide strategies that can be used by other scientific societies to hold similar events in their own communities.

## 1. Introduction

Ground-breaking scientific advances in hepatitis virology have been realised in recent decades. These have provided life-changing improvements for millions of people affected by viral hepatitis, particularly hepatitis B and hepatitis C (in the form of treatments and cures, respectively). Despite the outstanding pace of progress in the field, important problems still remain:

- Although current therapies can suppress hepatitis B virus (HBV) replication and reduce the risk of adverse outcomes, a finite-term therapeutic cure is still unavailable;
- There is no vaccine that protects against hepatitis C virus (HCV) infection or reinfection;
- The lack of rapid and available diagnostic tests for both infections remains a key obstacle to linkage to care;
- Access to information, treatment and care for people living with viral hepatitis is limited in many parts of the world;
- Stigma and discrimination against people with viral hepatitis limit their willingness to access health care and prejudices policies for community engagement, diagnosis, management, treatment and research funding.

These issues contribute to the ongoing new transmission events that occur every year, adding to the number of affected individuals. At the same time, these issues also define the leading edge of medical research, which may not be well publicised to the lay community. We aimed to communicate this scientific focus and progress to the general public (and particularly those affected by viral hepatitis) in an interactive panel discussion with experts in the field.

Here, we describe our event and provide potential strategies for others to convey the benefit of medical research, to successfully engage with affected communities and to improve researchers’ confidence in establishing their own outreach events.

## 2. Event Overview

An interactive discussion panel discussion was organised by the Australian Centre for Hepatitis Virology (ACHV), the key national society for hepatitis virology research in Australia. The event aimed to provide (1) a background on the history of scientific research in viral hepatitis and how it has improved the lives of those affected by viral hepatitis and (2) an opportunity for those from the affected community to have their questions answered by leaders in the field.

We invited experts from a broad range of backgrounds (from bench to bedside, in both HBV and HCV research) to be hosts and panel members (Table 1). This range of expertise provided several advantages:

1. The panel as a whole had a broader experience to call on to answer questions posed by the audience;
2. Given their non-overlapping expertise, the panel could ask each other questions to initiate constructive and informative conversations if the audience was not forthcoming with questions of their own;
3. The audience would see the diversity of input required for scientific advances and their translation from “bench to bedside”.

## 3. Event Organisation

### 3.1. Platforms for Pre-Event Organisation

The panel discussion event was fully virtual and freely accessible to anyone with an Internet connection globally. This allowed the greatest number of people to join and interact, particularly with extensive restrictions on travel and gatherings due to the COVID-19 pandemic. The event was held at 6 p.m. AEST (UTC + 10), making the time zone amenable to live attendance for people across most of the world’s regions.

Several platforms were employed to enhance participant experience and facilitate pre-event organisation. An Eventbrite (https://www.eventbrite.com.au/ (accessed on 29 July 2021, created on 8 July 2021)) page was set up for free registration, which enabled us to monitor enrolments in real time and send group emails to all participants (e.g., reminders, Zoom call details, etc.).

Registrants could ask questions either identifying themselves on the call or anonymously via Sli.do (https://app.sli.do/ (accessed on 29 July 2021, created on 8 July 2021)), an independent online platform for interactive discussions. On this platform, user-submitted questions can be up-voted by others, thus avoiding redundant questions. There were 11 questions posted on the Sli.do forum before the event (all but one were posted anonymously). During the live streaming, the majority of people asked questions via the Zoom chat function.

### 3.2. Advertising of the Event

**Figure 1:**
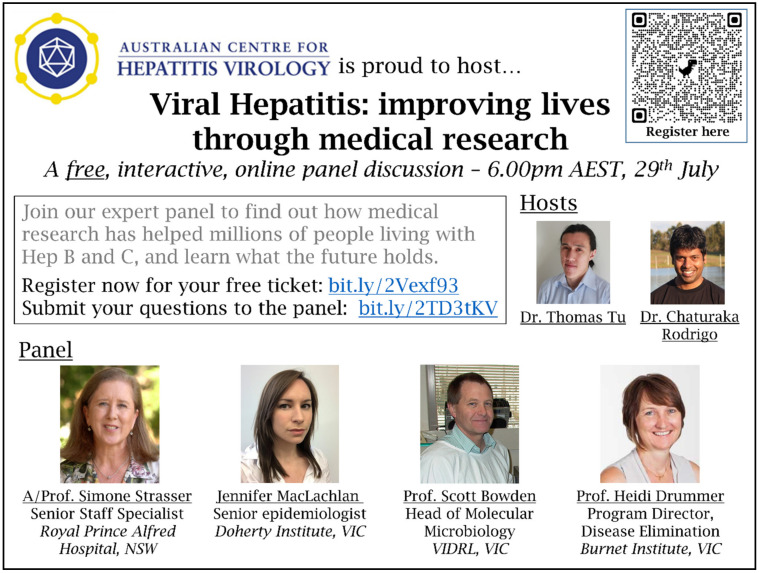
Poster designed by ACHV to advertise the discussion panel event.

Effective advertising was key to the success of the event, leading to a broad spectrum of registrants. We developed a poster to publicise the event and introduce the expert panel (Figure 1), which included a QR-code to the Eventbrite registration page and a URL to the Sli.do question submission page.

The poster was used to widely promote the event through professional networks, online patient forums, and social media. In addition to the ACHV membership base, we advertised the event through the Australasian Society for HIV, Viral Hepatitis and Sexual Health Medicine eNewsletter. In addition, we took advantage of World Hepatitis Day on 28 July 2021, the day before our event. We registered our panel discussion on the website developed by Hepatitis Australia (https://www.worldhepatitisday.org.au/ (accessed on 29 July 2021)) to centralise all national events focused on viral hepatitis taking place during the month.

The event was advertised on online platforms dedicated to the affected community chiefly by Dr. Thomas Tu (ACHV president) who openly lives with hepatitis B, adding trustworthiness and weight to the endorsement of the event. We targeted various social media platforms to gain broad awareness: Facebook, particularly groups visited by people with viral hepatitis (e.g., “Hepatitis B Foundation”, “Hepatitis B survivors”, “Hepatitis-B/C Support Group”—in total serving >30,000 members); Twitter, generating 1680 impressions; and LinkedIn, generating 828 views.

A steady increase in registrant numbers was observed after registration was opened about 2 weeks prior to the event. By the time of the event, 69 participants had registered (excluding the panel). The Eventbrite page had been viewed 579 times (12% of those viewing had registered), indicating a good conversion rate to registrations. Most of the traffic to the event page was directed through a link (424 of 579 total, 73.2%), indicating that promotion through links shared by us to professional and patient networks had a larger impact compared to people finding the event on the upcoming events on Eventbrite. The resultant audience was a mix of scientists, students, health professionals and general public (including people living with viral hepatitis).

## 4. The Event

The event was streamed on Zoom hosted by the University of Sydney on 29 July 2021. The entire event was recorded (informing participants prior to the event) and uploaded to YouTube (https://youtu.be/rVEnz4PtrEY (accessed and created on 30 July 2021)). The link was sent to each registrant via email regardless of whether they were able to join live to maximise impact and as an advertisement for future events.

### 4.1. Background Discussion

The discussion started with a moderated interview style discussion to provide an overview of the scientific advances in viral hepatitis and their effects on clinical practice. The discussion was pitched with a balance to cater to the needs of both the general public, as well as the affected community and other stakeholders who had more in-depth knowledge on the topic.

#### 4.1.1. Clinical History

A/Prof. Simone Strasser provided a brief overview of the clinical presentation of viral hepatitis and the state of clinical treatment for this condition in 1986 (the birth year of Dr. Thomas Tu, the event host and a person living with hepatitis B), providing the background context of the medical progress in the last few decades. During this early period, hepatitis B could only be treated with symptomatic management and supportive care. If the disease progressed to liver failure and was treated with liver transplantation, reinfection and loss of the graft was common, occurring in 80–100% and 50%, respectively [1]. As a result, many transplant programs around the world stopped offering liver transplantation to patients with chronic hepatitis B.

Prof. Scott Bowden (an HBV researcher during this time) reflected that this paucity of treatment options provided scientists with great impetus to improve patient lives. As fundamental Hepatitis B and C research was (and still remains) underfunded, the field instead turned to repurposing other antivirals (e.g., those used to treat HIV and herpes simplex virus). In Australia, the establishment of the duck model of HBV served as a testbed for activity against HBV replication [2,3,4,5]. Combined with pre-clinical studies, several agents with anti-HBV activity were discovered (e.g., famciclovir, used for treating herpes infections). While viral breakthrough and antiviral resistance were reported, additional agents were continually developed through this strategy (e.g., adefovir, lamivudine, tenofovir). The development of HBV antivirals was life-saving, not only allowing liver transplant patients to survive longer without graft reinfection, but also preventing disease progression to reduce the need for transplantation in the first place.

#### 4.1.2. Scientific Progress

Hepatitis C virus (HCV, the causative agent of what was then called non-A non-B viral hepatitis) had not even been discovered yet at this time. Prof. Heidi Drummer provided an in-depth recount of the Nobel Prize-winning work [6] of Harvey Alter (first clinical descriptions of non-A, non-B hepatitis [7,8]), Michael Houghton (isolation and identification of HCV [9]) and Charlie Rice (cloning of the genome and showing that it caused hepatitis [10]) in 1989.

After these keystone studies, thousands of scientists had worked on developing systems to grow HCV in the lab and develop drugs against it. In particular, the group of Ralf Bartenschlager developed a replicon system [11] that allowed high-throughput screening of millions of compounds, eventually leading to curative therapies for HCV. These have now directly transformed the lives of millions of people worldwide.

A large part of this progress has been due to earlier fundamental virology research, particularly the discovery of reverse transcription in retroviruses. Enzymes taken from these viruses enabled the initial identification of HCV and the cloning of its genome. Reverse transcription is also the sole target of all clinically used, direct-acting anti-HBV therapies. While not curative, these therapies can nevertheless suppress the virus and (as mentioned in the section above) have led to a dramatic increase in HBV patient survival.

Current research in the HBV field is now focused on curative therapies. Given the establishment of robust assays and model systems, new treatments are now being developed specifically for HBV, rather than repurposing drugs from other viral infections. The panel was hopeful that some of the investigational therapeutics will likely be curative in at least a subset of people.

#### 4.1.3. Broader Impacts

Jennifer MacLachlan, Head of the Australian National Hepatitis Mapping Project, then reflected on the community-wide and global trends associated with these medical discoveries. After the advent of curative HCV treatments and their funding through the Australian national health care system in 2016, epidemiological trends have shown a dramatic decrease in the number of people living with, newly acquiring and dying due to HCV infections [12,13]. These trends have been observed in other locations globally, but in many countries, access to HCV therapy has been limited [14]. Even in Australia, there has been a decline in treatment to below target levels, due in part to insufficient numbers of people receiving diagnosis and workup for treatment [15]. Moreover, despite the sharp reductions, new HCV infections continue to occur as there is no protective vaccine nor neutralising immunity after clearance of an infection.

As previously mentioned, reduced mortality has been observed since the introduction of highly effective treatments for HBV in Australia [16]. However, preventable deaths continue to occur as treatment uptake remains well below optimal levels [17], and the majority of people with hepatitis B in Australia are not engaged in care [18]. Improved engagement and support of communities affected by HBV, as well as increased access outside of tertiary hospital settings, will be essential to improving uptake. A limited term therapeutic cure for hepatitis B could lead to increased engagement in ongoing care, both from communities and from health providers. However, diagnosis and clinical assessment rates need to greatly improve if such a cure is to have substantial impact.

In summary, these trends emphasise the need to keep up momentum in the delivery of HCV treatment and expand engagement in HBV care and access to diagnosis for both conditions. It also highlights the importance of progress in the development of a protective vaccine for HCV infection and an effective HBV cure.

### 4.2. Question Time

The majority of the audience was highly engaged and keen to take the opportunity to query the expert panel. Participants were eager to provide personal perspectives and experiences and showed interest in asking queries about their own health experiences (e.g., when to start medication, why it is hard to receive medical treatment, potential side effects of treatment, how best to better one’s health, etc.). For this reason, we strongly recommend including an experienced clinician in the sub-speciality on the panel (in our case, A/Prof. Simone Strasser) to address these health-related questions.

Some queries and comments were raised about the inequitable accessibility of clinical treatment. Audience members from low- to middle-income countries raised how it is difficult to access specialist care in their country, the prohibitive costs of treatment and the fear of stigma preventing any access to support. These issues caught our Australia-based, academic-centric panel unprepared, given the lower barriers to health care in their own experienced. While it was helpful to have a person living with chronic hepatitis B to empathise with the audience’s situation, it also could have been an opportunity for an international health policy or public health expert to provide a hopeful and insightful response. ACHV is considering inviting people with this expertise for future panels.

In the end, a large influx of questions from the affected community was submitted both live and in the days leading up to the event. We had evenly split time between the background discussion and the Q&A session with the audience but in hindsight realise that the latter was much more engaging and in demand. In future events, we would likely alter the proportion of the event dedicated to audience participation (e.g., 1:2 background:discussion).

## 5. Improvements for Future Events

While this event was successful on the whole, we learnt several lessons to implement in future discussion panel events.

### 5.1. A More Focused Topic

Given the discussion, the panel felt that the approach in describing the history of scientific breakthroughs in both HBV and HCV was likely too broad and had too many important points over too many areas to be clearly understood or recalled by the non-specialist public. Provided resources are available, ACHV now intends to organise quarterly events and provide a more in-depth discussion on more specific topics.

### 5.2. A More Positive Advertising Campaign

**Figure 2:**
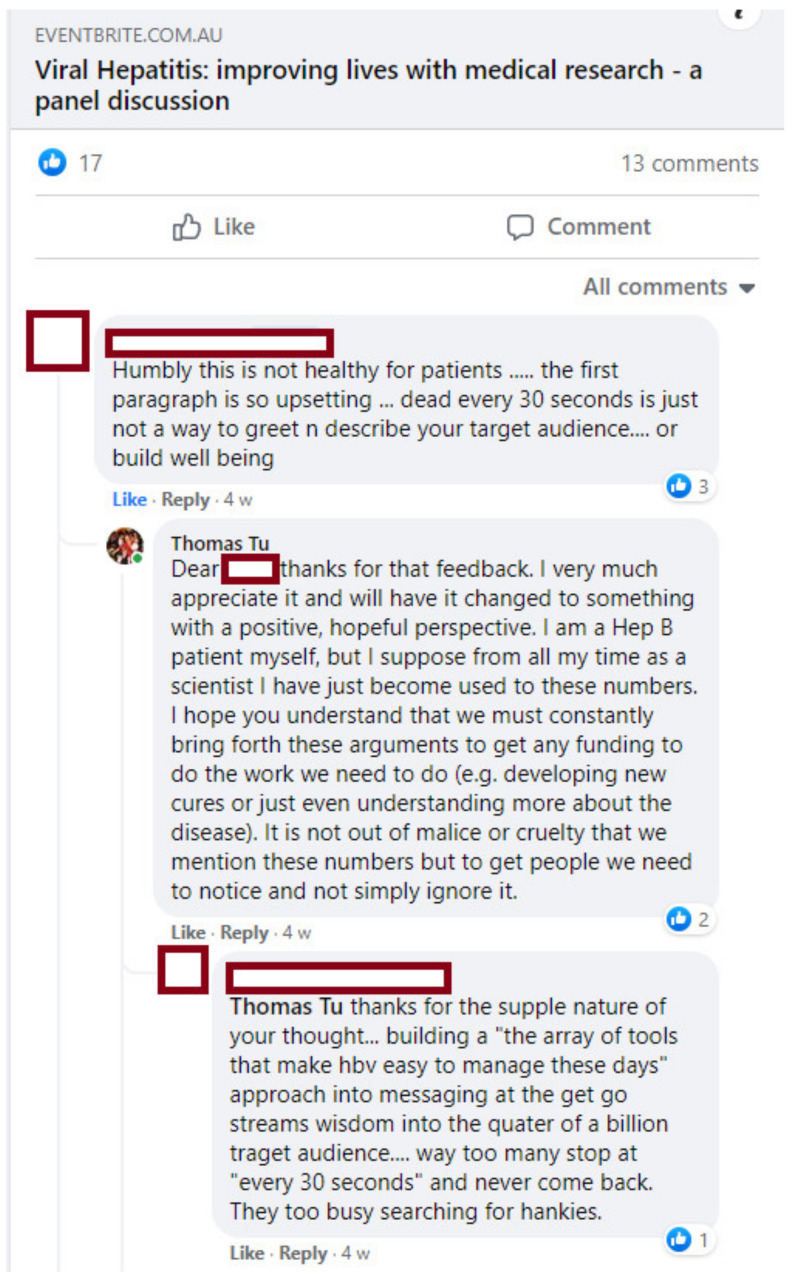
Facebook conversation about the event description and its unsuitability for the affected community.

Given the academic and biomedical background of the organising committee, the description of the event on the EventBrite page had highlighted the importance of viral hepatitis and its impacts on mortality and morbidity. After a Facebook conversation about the EventBrite page (Figure 2), it became clear that some people from the affected community were put off by excessive emphasis on the deaths associated with viral hepatitis.

While we had known to some extent that the fear of disease progression and death plays a significant role in the impacts of viral hepatitis [19], we had not foreseen this poor response. In response to this feedback, we promptly replaced the mortality statistics with a more hopeful message focused on how medical research has improved and continues to improve the lives of people with viral hepatitis. We encourage others running their own events to convey a positive perspective and to listen to feedback from the affected community (or even consult with them prior to publicising).

### 5.3. Reflection Session

During the discussion, panel members were reminded that much of the cutting-edge scientific progress is far removed from the experiences of the people living with viral hepatitis. Outside of Australia and the rarefied environment of academia/tertiary clinics, many people still struggle to access basic health care, diagnostic tests and antiviral treatment.

In future events, we will consider adding a section for the panel to discuss what they have learnt during the discussion. We believe that it would be empowering for the community to know that they are influencing and informing experts (as well as the other way around).

## 6. Final Thoughts and Conclusions

Overall, the expert panel discussion was a successful event by several measures. We increased the awareness of both our society and partner groups, including HepBCommunity.org, a publicly available online forum specifically designed to link people with hepatitis B with each other and experts in the field (founded and directed by Dr. Thomas Tu). This site provides a gateway to ongoing engagement and education in viral hepatitis including that which we provided on the night. This effective outreach to affected communities is key to ongoing public health measures and public support for scientific research. Our experience also showed that, provided the opportunity, many from the affected community are willing to engage and that some had even planned on starting community-based organisations themselves.

We would encourage other societies around the world to conduct similar events to broaden the access to informed experts in different countries, languages and disease conditions. Our event was limited by being held in English (which the majority of people with viral hepatitis do not speak) and had limited expertise on the conditions outside of Australia. However, there is clearly demand for educational content such as this in the community.

Communities that are more engaged with medical scientists are more likely to understand how public health decisions are being made. We feel that this is particularly important during this volatile period of increasing anti-expert and anti-science rhetoric. We believe that with these increased dialogues and respectful partnerships, researchers can play a more active role in society and its improvement.

## Figures and Tables

**Table 1:** Event hosts and panel members.

Name	Role	Relevant Positions Held ^1^	Specialty Area
Dr. Thomas Tu	Host	Group Leader—Viral Hepatitis Pathogenesis (WIMR) Founder and Director (HepBcommunity.org) President (ACHV)	Basic virology—HBV Patient advocacy Lived experience of hepatitis B
Dr. Chaturaka Rodrigo	Host	Group Leader—Viral Immunology Systems Program (Kirby Institute) Secretary (ACHV)	Basic virology—HCV
Prof. Heidi Drummer	Panel member	Program Director, Disease Elimination (Burnet Institute) Scientific Director, Burnet Diagnostics Initiative (BDI) Past president (ACHV)	Basic virology—HCV
Prof. Scott Bowden	Panel member	Former Head of Molecular Microbiology (VIDRL) Former senior scientist (WHO Reference HBV Laboratory) Past executive member (ACHV)	Basic virology—HBV Testing policy
Jennifer MacLachlan	Panel member	Head of National Viral Hepatitis Mapping Project (Doherty Institute)	Epidemiology
A/Prof. Simone Strasser	Panel member	Senior Staff Specialist, AW Morrow Gastroenterology and Liver Centre (RPAH) Director of Hepatology Clinical Trials (RPAH) President (GESA)	Clinical practice Clinical trials

^1^ WIMR, Westmead Institute for Medical Research; ACHV, Australian Centre for Hepatitis Virology; BDI, Burnet Diagnostics Initiative; VIDRL, Victorian Infectious Diseases Reference Laboratory; RPAH, Royal Prince Alfred Hospital; GESA, Gastroenterological Society of Australia.
